# High-intensity interval exercise affects neurogenesis and angiogenesis differently in diabetic and non-diabetic mice

**DOI:** 10.3389/fnins.2026.1927709

**Published:** 2026-08-20

**Authors:** Harald S. Mjønes, Mona Havik, Gezime Seferi, Cecilie Morland

**Affiliations:** Section for Pharmacology and Pharmaceutical BioSciences, Department of Pharmacy, Faculty of Mathematics and Natural Sciences, University of Oslo, Oslo, Norway

**Keywords:** angiogenesis, *db/db*, exercise, hippocampus, neurogenesis, type 2 diabetes

## Abstract

**Introduction:**

Exercise and metabolic health are key modulators of brain plasticity, influencing processes such as angiogenesis and neurogenesis.

**Methods:**

The present study investigated the effects of eight weeks of high-intensity interval training on capillary density and neurogenesis in the hippocampus of diabetic (*db/db*) and non-diabetic (*db/^+^*) mice, using established markers for capillaries (collagen IV) and immature neurons (doublecortin) through a series of immunolabelling experiments on fixed brain tissue.

**Results:**

We show that exercise significantly enhanced capillary density in the dentate hilus of diabetic mice, while having no effect in non-diabetic mice. Conversely, neurogenesis in the subgranular zone was increased by exercise in non-diabetic mice but remained unaffected in their diabetic littermates. Furthermore, correlation analyses challenge the dogma that angiogenesis directly promotes neurogenesis. We found no effect of either exercise or genotype on capillary density in the stratum radiatum or neurogenesis in the ventricular-subventricular zone, indicating a regional specificity of these processes.

**Discussion:**

These findings challenge and nuance established concepts of cerebral angiogenesis and adult neurogenesis, and their regulation in response to T2D and physical exercise Understanding these differential responses to exercise provides new insights into cerebral effects of diabetes and may lay the foundation for tailoring interventions to optimize brain health in individuals with metabolic disorders.

## Highlights

Exercise and metabolic health modulate hippocampal angiogenesis and neurogenesis.

High-intensity interval training enhances capillary density in the dentate hilus of type 2 diabetic mice but not in their non-diabetic littermates, indicating a distinctive response of diabetic brains upon exercise.

High-intensity interval training enhances neurogenesis in the subgranular zone in non-diabetic mice but not in their diabetic littermates.

These findings suggest that type 2 diabetes mellitus modulates the effects of exercise on the adult brain, providing insights into the cerebral effects of diabetes, and possibly opening for customized interventions to enhance brain health in individuals with metabolic disorders.

## Introduction

Type 2 diabetes mellitus (T2D) is a major metabolic disorder characterized by hyperglycemia, insulin resistance, and β-cell dysfunction. Beyond peripheral metabolic disruptions, T2D is associated with structural and functional alterations in the brain. Increasing evidence indicates that T2D negatively affects the hippocampus, a region essential for learning, memory, and mood regulation. Impaired cerebral perfusion, vascular integrity, and angiogenesis appear to contribute to this effect, and are linked to cognitive decline (Biessels and Reagan, 2015; Cheng et al., 2018; Forouhi and Wareham, 2010). For instance, humans with T2D exhibit reduced blood flow in multiple brain regions, including the hippocampus and several cortical areas (Cui et al., 2017), resembling what is seen in early dementia and correlating with insulin resistance and poorer cognition (Bangen et al., 2018; Cui et al., 2017). Furthermore, animal models of T2D show abnormal vessel remodeling and features of pathological neovascularization (Prakash et al., 2012, 2013) as well as compromised blood-brain barrier (BBB) integrity (Frank and McNay, 2022; Nuthikattu et al., 2024). Some of these features could be alleviated by treatment with the anti-diabetic drug metformin, suggesting hyperglycemia as a mediator of pathological neovascularization in T2D. Adult neurogenesis is also negatively affected by T2D, as well as by ageing and dementia (Bonds et al., 2020; Zaletel et al., 2018).

Physical exercise is among the most robust non-pharmacological interventions known to promote brain health. In humans as well as in experimental animal models, exercise improves systemic metabolic control while also stimulating hippocampal plasticity, including neurogenesis and angiogenesis (Erickson et al., 2011; Hillman et al., 2008; Pereira et al., 2007; van Praag et al., 1999; Voss et al., 2019). Two reviews with meta-analyses summarizing data from 71 to 44 randomized control trials (number of participants 5606; 4793, respectively), of persons with cognitive decline or dementia concluded that several types of exercise decelerate cognitive decline (Gallardo-Gómez et al., 2022). Interestingly, the latter study reported a dose-dependent effect of exercise on cognitive functions in older adults (Gallardo-Gómez et al., 2022).

Exercise paradigms differ markedly in intensity and metabolic demand, and high-intensity interval training (HIIT) has emerged as a potent strategy for improving glucose homeostasis and cardiovascular fitness, with growing interest in its effects on the brain (Cassidy et al., 2017; Saucedo Marquez et al., 2015). We have previously shown that HIIT is more effective than medium-intensity interval exercise (MIIT) at promoting neurogenesis in the subgranular zone (SGZ) and subventricular zone (SVZ) of non-diabetic mice and that SVZ neurogenesis is regulated by lactate signaling through HCAR1 (Lambertus et al., 2021, 2024). Similarly, exercise is a known inducer of angiogenesis in the brain (Yoon et al., 2023; Zang et al., 2023), and we have previous demonstrated that HIIT induces HCAR1-dependent angiogenesis in the hippocampus as well as in the sensory-motor cortex; an effect which could be mimicked by lactate injections via activation of HCAR1 (Morland et al., 2017).

T2D may fundamentally alter the brain’s adaptive response to exercise. Chronic hyperglycemia, low-grade inflammation, insulin signaling deficits, and endothelial dysfunction can disrupt the neurovascular niche that supports precursor cell proliferation and differentiation. Studies in diabetic rodents have reported reduced hippocampal cell proliferation and impaired vascular integrity, suggesting that the diabetic milieu may constrain exercise dependent plasticity (Beauquis et al., 2006; Ho et al., 2013; Stranahan et al., 2008; Targett et al., 2026). Previous studies on the *db/db* mouse model have reported that aerobic moderate-intensity continuous training (MICT) alleviates depressive-like symptoms and cognitive impairment (Zhou et al., 2022), while HIIT was reported to have better effect on glucose metabolism than MICT (Chavanelle et al., 2017). Whether HIIT-induced vascular and neuronal plasticity is preserved in the T2D brain is largely unresolved and this knowledge could inform targeted rehabilitation strategies to prevent or slow diabetes-related cognitive decline.

In the present study, we randomized diabetic *db/db* mice–a model of T2D–and their non-diabetic *db/^+^* littermates to 8-weeks of HIIT or sedentary conditions. Using well-established immunohistochemical markers for blood vessels and neurogenesis we examined whether HIIT differently regulated brain capillary density and neurogenesis in the two genotypes. Since adult neurogenesis often occurs near newly formed capillaries, we also assessed whether capillary density in the dentate hilus or stratum radiatum correlated with neurogenesis in the adjacent SGZ.

## Materials and methods

### Animals

All animal experiments were approved by the Norwegian food safety agency (FOTS ID: #21282) *a priori* and were conducted in strict accordance with national and institutional guidelines and the Directive 2010/63/EU. The experiments are reported according to the ARRIVE (Animal Research: Reporting of *In Vivo* Experiments) guidelines 2.0 (Percie du Sert et al., 2020). Animals belonging to the B6.BKS(D)-Leprdb/J strain were bought from The Jackson Laboratory (RRID:IMSR_JAX:000697) and bred in-house at the Department of Comparative Medicine at the Faculty of Medicine, University of Oslo. A mutation in the *Lepr* gene results in a defect leptin receptor, which renders the homozygote (*db/db*) mice unable to respond to leptin. Consequently, the mice overeat, become obese and develop T2D from around 8 weeks of age on a regular diet (Coleman, 1978; Dalbøge et al., 2013; The Jackson Laboratory, 2009). Heterozygote animals (*db/^+^*) produce enough of the functional leptin receptors to not develop the pathological phenotype. Hence, *db/^+^* littermates of the *db/db* mice were used as non-diabetic controls in the present study, as advised by the Jackson Laboratory (Coleman, 1978; The Jackson Laboratory, 2009). Using the heterozygotes littermates as control animals minimized genetic variation between groups and was, together with all other aspects related to animal handling (see below), done to avoid confounding factors. After weaning, the animals were kept in Green Line Sealsafe Plus GM500 or GR900 cages (Techniplast, Italy). Each cage held a maximum of five mice, separated by sex, but irrespective of genotype or exercise status (described below). Animals had continuous access to chow and water, and each day was divided into 12 h–12 h light-dark cycles. The cages did not have running wheels but were enriched with a small plastic house or chewable tunnels, and a wood gnawing stick.

A total number of 36 mice were included in the study. To apply with the three Rs principle (replacement, reduction and refinement), and reduce the number of animals used in research, the analyses presented in the present study are from the same animals as in a recent paper from our group (Seferi et al., 2024). Hence, the methods for genotyping, exercise, and tissue preparation were described in the previous paper, and presented again in the present manuscript for completeness and readability.

At 6 weeks of age, the mice were ear marked for identification. The ear biopsies from the identification marking were used for genotyping by using the GenElute*™* Mammalian Genomic DNA Miniprep Kit (Sigma-Aldrich, G1N350-1KT). The protocol was provided by the manufacturer. The samples were subjected to tetra-primer amplification refractory mutation system-polymerase chain reaction using two pairs of primers to amplify two alleles, one wild-type allele (G allele) and one mutant allele (T-allele). The protocol was based on the study by Peng et al. (2018). The primers used for amplification from normal and mutant leptin receptor alleles were forward outside primer: 5′-TTGTTCCCTTGTTCTTATACCTATTCTGA-3′; reverse outside primer: 5′-CTGTAACAAAATAGGTTCTGACAGCAAC-3′; forward inner primer: 5′-ATTAGAAGATGTTTACATTTTGATGGAAGG-3′; reverse inner primer: 5′-GTCATTCAAACCATAGTTTAGGTTTGTCTA-3′.

### Open field test

All animals were subjected to an open-field test prior to the exercise regimen. After habituation in the experimental room for 1 h, animals were placed in separate 40 × 40 cm arenas (VersaMax Animal activity monitor, AccuScan Instruments, Inc., USA). The experiment room was silent and the light within each arena was measured and confirmed to be between 38 and 41 lux. Research personnel left the room during the test while infrared sensors on the floors of the arenas tracked the animal’s movements for 1 h. The test was carried out separately for female and male mice, with the females being tested first.

### Exercise

At ages between 9 and 11 weeks, animals belonging to the diabetic *db/db* genotype, or the non-diabetic *db/^+^* genotype were randomized to either participate in the exercise regime or remain sedentary. Mice in the exercise group underwent HIIT on treadmills (Exer 3/6 Treadmill, Columbus Instruments, USA) at a 25-degree incline. The mice exercised for 5 consecutive days, followed by 2 rest days, for a total of 8 weeks. This exercise regimen is 1 week longer but other ways equal to that described in Morland et al. (2017) and is designed to make the animals reach approximately 90% VO_2max_ and ensure ideal cardiovascular function. During the first exercise session, the running speed was gradually increased to the appropriate speed as the animals familiarized themselves to the treadmill running. A maximum endurance capacity test (MECT) was carried out every second Tuesday during the intervention period. The results from the MECT were used to adjust the running speed for the next week to ensure appropriate exercise load throughout the 8 weeks [see Morland et al. (2017)], but the running speed was also increased during each interval session and throughout the 2 weeks between the MECTs based on the observed performance of the mice.

Each ordinary exercise day started with a 10-min warm-up, where the running speed was set to 8 m/min for the *db/^+^* animals and 5 m/min for the *db/db* animals. This difference in warm-up speed was implemented to ensure that the warm-up remained a genuinely low-intensity phase for both genotypes, as the maximal running capacity of the *db/db* mice was only slightly above 8 m/min at the start of the intervention. The animals ran 10 × 4-min intervals with a 2-min rest between each interval. The treadmill allows six mice to exercise simultaneously. The mice ran in individual lanes, separated by walls, but on the same belt. Hence, the running speed was set to approximately 80% of the average MECT result for the animals who ran on the same belt. Based on observations during the interval session, some animals occasionally received 10-s breaks if needed. The following exclusion criteria were set *a priori*: If an individual mouse performed worse than expected (based on the previous performance of the same mouse) and appeared to struggle even when given additional 10-s-breaks, it was withdrawn from the rest of that exercise session. If the same animal performed worse than expected on two consecutive running sessions, it was presumed injured or sick and withdrawn from the study. During the experiment, the animals’ body composition and weight were measured weekly (Seferi et al., 2024), and any sign of discomfort was observed for on a daily basis. If a mouse showed signs of discomfort (stereotypic behavior, weight loss, or fur changes) it would be excluded from the study. None of the animals met these exclusion criteria, but one *db/db* mouse was excluded from the exercise regimen as it failed to develop an obese phenotype. Of the remaining 35 animals, 14 belonged to the *db/^+^* sedentary group (8 males, 6 females), 6 belonged to the *db/^+^* exercised group (3 males, 3 females), 10 belonged to the *db/db* sedentary group (5 males, 5 females), and 5 belonged to the *db/db* exercised group (2 males, 3 females).

### Tissue preparation

At 6 h after the last exercise session, the mice were deeply anesthetized by intraperitoneal injections of 10 μL/g bodyweight of ZRF-mix [zolazepam (3.3 mg), tiletamine (3.3 mg), xylazine (0.5 mg), and fentanyl (2.6 μg/mL)]. A sufficient level of anesthesia was verified by the toe pinch test at 10–15 min after injection of the ZRF-mix. Then, the animals were transcardially perfused with room-tempered paraformaldehyde (PFA, 4%) in 0.1 M sodium phosphate buffer (NaPi, pH 7.4). The fixative was freshly prepared and filtered before each day of use and was injected via a cannula inserted in the left ventricle of the heart. A peristaltic pump connected to the cannula administered the fixative systematically via the circulation at a pace equal to the cardiac output of mice, for 8 min. All exercised animals were perfused within approximately 50–60 min and the sedentary mice were perfused immediately after the exercised mice to minimize physiological variation due to the time-of-day (Nelson et al., 2021). Brains were then gently removed from the skull and placed in 4% PFA over-night. On the following day, the fixative was replaced with 0.4% PFA in which the brains were stored at 4 °C until cryoprotection and sectioning.

For cryoprotection, the brains were immersed in 30% sucrose in 0.1 M NaPi (pH 7.4) between 12 and 24 h before sectioning. An HM450 sliding freezing microtome (Thermo Scientific, Walldorf, Germany) was used to slice the brain into 20 μm thick sagittal sections. Sections were then stored in 0.1 M NaPi buffer (pH 7.4), containing 0.02% sodium azide at 4 °C.

### Immunohistochemistry

Brain sections were chosen approximately 1.7 mm lateral to the longitudinal fissure (Allen Institute for Brain Science, 2008). Prior to immunolabeling for the basal lamina marker collagen IV, the brain sections were mounted on glass slides (Superfrost^®^ Plus, Thermo Scientific, Germany/USA) and allowed to dry. A PAP-pen (Liquid Blocker, Ref. No. 71310, Electron Microscopy Sciences, USA) was used to draw a square along the edges of the glass slide. For epitope retrieval, a pepsin solution (1 mg/mL pepsin (ref: 10108057001, lot: 39655522, Roche, Switzerland) dissolved in 0.2 M HCl) was pipetted onto the brain sections and the slides were placed in the heating cabinet (36.5 °C) for 16 min. Sections were then rinsed with phosphate buffer saline (PBS, pH 7.4) for 3 × 10 min, before incubation with a blocking solution containing 3% newborn calf serum (NCS), 0.5% bovine serum albumin (BSA), and 0.5% Triton X-100 in PBS, for 2 h. The sections were then incubated with anti-collagen IV antibodies (Ab 6586, lot: GR3350938-18, ABCAM, USA, RRID:AB_305584), diluted 1:500 in blocking solution, on a gentle shaker at room temperature overnight. Sections were then rinsed in PBS 6 × 10 min, before incubation with an Alexa Fluor™ donkey anti-Rabbit 488 secondary antibody (lot: 187477, Invitrogen, USA, RRID:AB_2535792), diluted 1:1000 in blocking solution, for 2 h in the dark. Then, the sections were rinsed 3 × 10 min in PBS, before a 15-min incubation in 4’,6-diamidino-2-phenylindole (DAPI) diluted 1:5000 in PBS (D1306; Invitrogen). A final 3 × 10-min rinse in PBS was performed, before the sections were allowed to dry, mounted with ProLong™ Gold (ref: P36930, lot: 2626504, Invitrogen, USA) and cover slips (Menzel Glaser, Thermo Scientific, Germany/USA). The slides were kept in the dark at 4 °C until imaging.

Immunolabeling of neuronal progenitor cells and migrating immature neurons was performed on free-floating sections. The sections were placed in 24-well plates (TC Plate 24 Well, Standard, F, Sarstedt AG and Co., Germany, ref: 83.3922.005), one section per well. First, the sections were rinsed 2 × 5 min in PBS, prior to antigen retrieval in 0.01 M citrate buffer (pH 8.6) at 80 °C for 30 min. The subsequent rinsing, and incubation steps were performed as described for the labeling with collagen IV, but the following combination of primary and secondary antibodies was used: anti-Doublecortin (DCX) (AB2253, lot: 3951142, Merck Millipore, USA; produced in guinea pig, RRID:AB_1586992), diluted 1:500, followed by Alexa Fluor™ goat anti-guinea pig 594 (lot: 2160074, Invitrogen, USA, RRID:AB_2534120) secondary antibody, diluted 1:500. Finally, the sections were mounted on microscopy slides (Superfrost^®^ Plus, Thermo Scientific, USA) and cover slips were attached (Menzel Glaser, Thermo Scientific, Germany/USA) with ProLong™ Gold (ref: P36930, lot: 2626504, Invitrogen, USA). The slides were kept in the dark at 4 °C until imaging.

### Imaging

Fluorescence z-stack images of whole-brain sagittal sections at a resolution of 0.11 μm per pixel were obtained at the Norbrain Slidescanning Facility^1^, using the AxioScan Z1 (Carl Zeiss) at the Institute of Basic Medical Sciences (Faculty of Medicine, University of Oslo).

### Image analysis

Maximum intensity projection (MIP) files of the scanned brain sections labeled with anti-Collagen IV were analyzed semiautomatically in Fiji (ImageJ, version 2.1.0, USA), with assistance from the Trainable WEKA Segmentation plugin (Arganda-Carreras et al., 2017). All analyses were performed by an observer who was blinded regarding intervention and genotypes. The area of the hilus of the dentate gyrus was outlined as the area between the inner borders of the granule cell layer and a straight line between the two extremes of the dentate granule cell layer. A classifier was trained to recognize collagen IV-labeled blood vessels within this area and exclude the background. Vessels larger than 10 μm in diameter were considered to be arterioles/arteries or venules/veins and excluded from further analysis. The area of these larger vessels was subtracted from the total area of brain tissue analyzed to yield a final region of interest (ROI) in which capillary density was measured. Capillary density for each animal was calculated as follows: the total area of capillaries (μm^2^) was divided by the final ROI area (μm^2^). The numbers for each individual were then normalized to the average of the control group (*db/^+^* sedentary) and the statistical comparison between the groups was done on the normalized data.

Neurogenic cells in brain sections treated with anti-DCX were initially counted manually along the SGZ, and the density of DCX^+^ cells for each animal was calculated as the number of DCX^+^ cells divided by length of the granule cell layer in μm. However, counting DCX^+^ cells turned out to be challenging, as dendritic projections from positive cells may surround adjacent nuclei, making it difficult to judge whether these adjacent cells are in fact DCX^–^ or DCX^+^. This problem was especially apparent in the V-SVZ, where manual counting turned out to be impossible. To overcome this problem, we measured the area of the positive immunosignal instead of counting each cell. By using the “oval selection” tool in Fiji, the mean gray value of the background was calculated. Background was measured in the dentate hilus or the striatum and subtracted from the image before measuring the area of positive cells in the SGZ or SVZ, respectively. The “polygon selection” tool was then used to outline the granular cell layer of the dentate gyrus and the cell layer in the V-SVZ, based on the DAPI signal. A classifier from the trainable WEKA segmentation plugin (Arganda-Carreras et al., 2017) was trained to recognize DCX^+^ signal in each of these areas. The density of DCX^+^ cells for each animal was estimated by dividing the area of DCX^+^ signal (μm^2^) by the length of the SGZ or the SVZ (μm), respectively.

A linear regression model was created in order to compare results from the two methods. This model demonstrated that an increase in number of DCX^+^ cells/μm in the SGZ correlated with an area of DCX^+^ signal/μm in the SGZ (R^2^ = 0.68, *p* < 0.0001, GraphPad Prism; Supplementary Figure 1). An R^2^ of 0.68 shows that the overall trend is similar between methods. The unexplained variation is presumably due to the imprecise counting of DCX^+^ cells.

### Statistics

All statistical analyses were performed in GraphPad Prism (version 10). The significance level was 5%. Prior to statistical analyses the data were normalized by dividing each value by the mean value of the sedentary *db/^+^* mice. Statistical outliers were removed if they were outside the 1.5 × interquartile range. Normality of residuals was checked with qq-plots and the Shapiro-Wilk test. Two-way ANOVA, followed by a Tukey’s *post hoc* test were used to analyze for differences between the groups in datasets where normal distribution and equal variance could be assumed. A student’s *t*-test or paired *t*-test was used appropriately when comparing two groups, in datasets with normal distribution and equal variance. Data that did not pass the Shapiro-Wilk test, were log2-transformed prior to statistical analysis. The Mann-Whitney U test was applied for the dataset belonging to the first 10 min of the open-field test, as log2 transformation did not yield normal distributed data. Results are shown as mean ± standard deviation (SD), unless otherwise specified.

## Results

### Capillary density of the dentate hilus was enhanced by exercise in diabetic mice but not in non-diabetic mice

**FIGURE 1:**
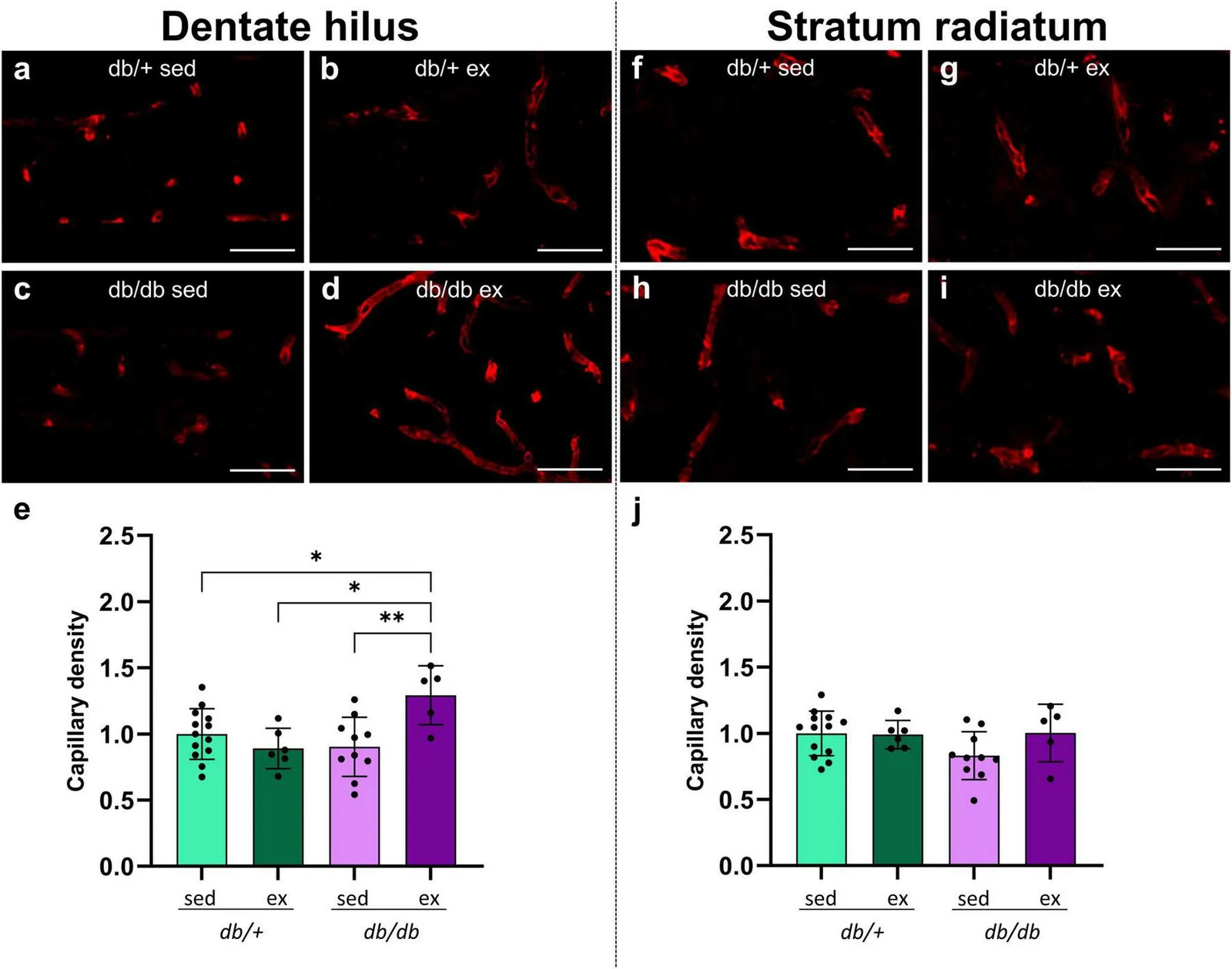
Effects of exercise on capillary density in the dentate hilus and stratum radiatum of diabetic and non-diabetic mice. **(a–d)** Maximum intensity projection images of scanned brain sections labeled for the basal lamina marker collagen IV (red) in the dentate hilus. **(a)** *db/^+^* sedentary (*db/^+^* sed). **(b)** *db/^+^* exercised (*db/^+^* ex). **(c)** *db/db* sedentary (*db/db* sed). **(d)** *db/db* exercised (*db/^+^* ex). **(e)** Bar plot showing mean capillary density in the dentate hilus for each group, normalized to *db/^+^* sed. *db/^+^* sed (light green), *db/^+^* ex (dark green), *db/db* sed (light purple) and *db/db* ex (dark purple). Dots represent observations from individual animals. Error bars indicate the SD. **(f–i)** Maximum intensity projection images of scanned brain sections labeled for the basal lamina marker collagen IV (red) in the stratum radiatum. **(f)** *db/+* sedentary. **(g)** *db/+* exercised. **(h)** *db/db* sedentary. **(i)** *db/db* exercised mice. **(j)** Bar plot showing mean capillary density in the stratum radiatum for each group, normalized to the *db/+* sedentary group. *db/+* sedentary (*db/+* sed; light green), *db/+* exercised (*db/+* ex; dark green), *db/db* sedentary (*db/db* sed; light purple) and *db/db* exercised (*db/db* ex; dark purple). Dots represent observations from individual animals. Error bars indicate the SD. Number of mice (n): 13 *db/^+^* sed, 6 *db/^+^* ex, 10 *db/db* sed, *db/db* ex. *p ≤ 0.05, **p ≤ 0.01. Scale bars: 25 μm.

The capillary density in the dentate hilus was not affected by the obese/diabetic phenotype; capillary densities (normalized to the mean of the sedentary *db/^+^* mice) were 1.0 ± 0.19 in sedentary *db/^+^* mice vs. 0.9 ± 0.22 in sedentary *db/db* mice (*p* = 0.66; Figures 1a vs. 1c, e). Surprisingly, exercise did not affect the capillary density of the *db/^+^* mice, as exercised *db/^+^* mice showed capillary densities (0.89 ± 0.15) that were similar to those found in their sedentary counterparts (1.0 ± 0.19; *p* = 0.69; Figure 1a vs. 1b, 1e). A strong interaction effect between the genotype and the intervention was, however, found (*p* = 0.002), and this mainly reflected an increase in capillarization in the diabetic mice in response to exercise: In the *db/db* mice, exercise induced a 43% increase in capillary density of the dentate hilus, reaching 1.29 ± 0.22 (*p* = 0.007; compared to the sedentary *db/db* mice; Figure 1c vs. 1d, 1e). The capillary density of the dentate hilus of the exercised diabetic (*db/db*) mice was also 45% higher than in the exercised non-diabetic (*db/^+^*) mice (*p* = 0.01) and 29% higher than in the non-diabetic sedentary control (*db/^+^*) mice (*p* = 0.04).

### Capillary density of the stratum radiatum was unaffected by exercise in both diabetic and non-diabetic mice

The capillary density in the stratum radiatum of the *db/^+^* sedentary control mice (normalized to the capillary density in the dentate hilus) was 0.63 ± 0.11, which was lower than in the dentate hilus (1.00 ± 0.19; *p* < 0.0001; paired student’s *t*-test). The mean capillary density in stratum radiatum was not affected by the genotype or the exercise intervention, or by the interaction between the two. After normalizing the data to the *db/^+^* sedentary control group in stratum radiatum, the capillary densities in the four groups of mice were as follows: *db/^+^* sedentary 1.00 ± 0.17, *db/^+^* exercised 0.99 ± 0.11, *db/db* sedentary 0.83 ± 0.18, *db/db* exercised 1.00 ± 0.22 (Figures 1f–j).

### Exercise increases neurogenesis in the SGZ of non-diabetic mice but not in diabetic mice

**FIGURE 2:**
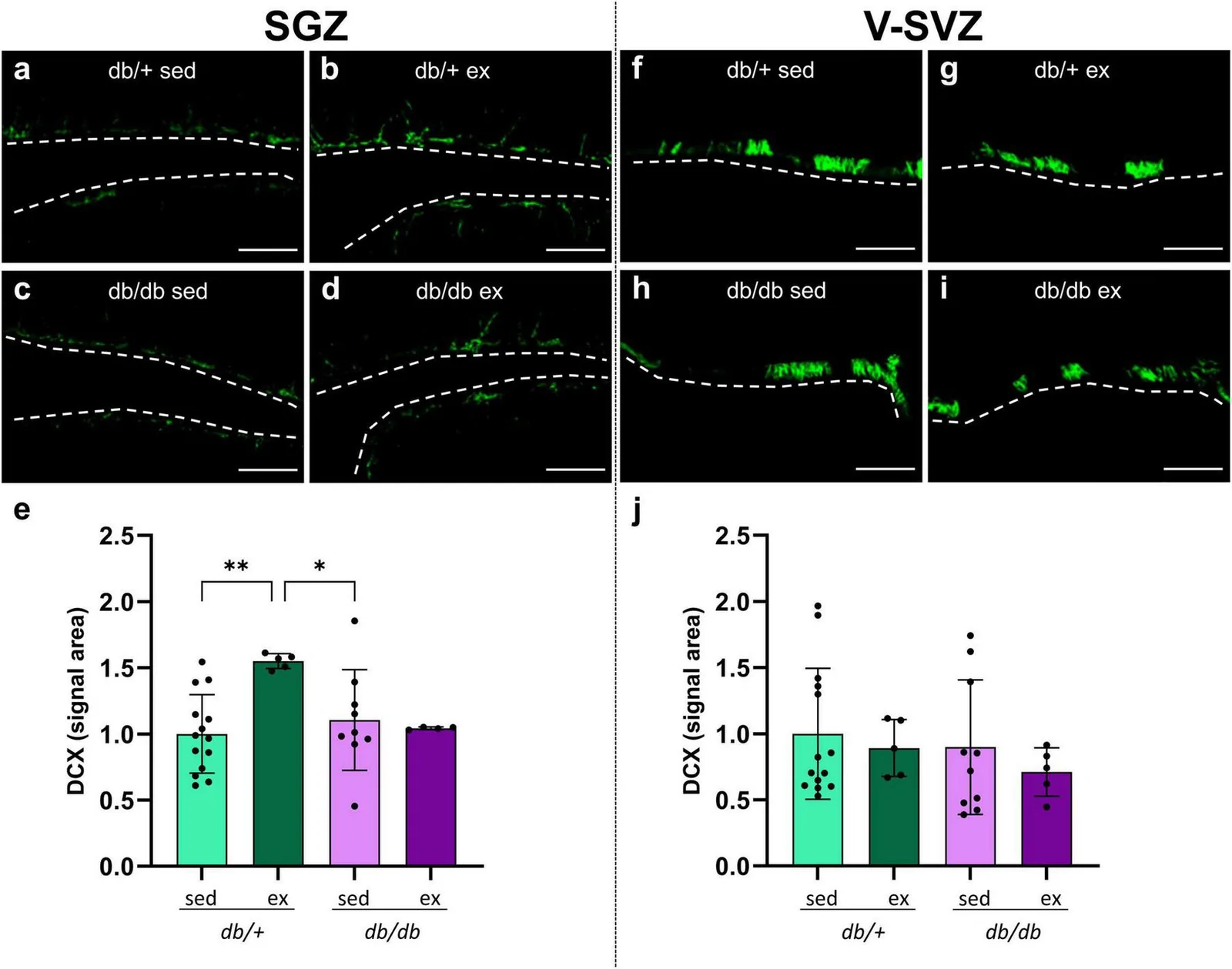
Effects of exercise on neurogenesis in the subgranular zone and ventricular-subventricular zone in diabetic and non-diabetic mice. **(a–d)** Maximum intensity projection images of scanned brain sections labeled for doublecortin (DCX), a marker for neuronal progenitor cells and migrating immature neurons (green) in the subgranular zone (SGZ). **(a)** *db/^+^* sedentary (*db/^+^* sed). **(b)** *db/^+^* exercised (*db/^+^* ex). **(c)** *db/db* sedentary (*db/db* sed). **(d)** *db/db* exercised (*db/db* ex). **(e)** Bar plot showing mean doublecortin (DCX) signal area in the subgranular zone (SGZ) for each group, normalized to the *db/^+^* sedentary group. *db/^+^* sed (light green), *db/^+^* ex (dark green), *db/db* sed (light purple) and *db/db* ex (dark purple). Dots represent observations from individual animals. Error bars indicate the SD. Number of mice (n): 14 *db/^+^* sed, 5 *db/^+^* ex, 9 *db/db* sed, 4 *db/db*. **(f–i)** Maximum intensity projection images of scanned brain sections labeled for doublecortin (DCX; green) in the ventricular-subventricular zone (V-SVZ). **(f)** *db/^+^* sed. **(g)** *db/^+^* ex. **(h)** *db/db* sed. **(i)** *db/db* ex. **(j)** Bar plot showing mean doublecortin **(DCX)** signal area in the stratum radiatum for each group, normalized to the *db/^+^* sedentary group. *db/^+^* sed (light green), *db/^+^* ex (dark green), *db/db* sed (light purple) and *db/db* ex (dark purple). Dots represent observations from individual animals. Error bars indicate SD. n: 14 *db/^+^* sed, 5 *db/^+^* ex, 10 *db/db* sed, 5 *db/db* ex. *p ≤ 0.05, **p ≤ 0.01. Scale bars: 50 μm.

Neurogenesis in the SGZ was not affected by the obese/diabetic phenotype *per se*, as the DCX signal area was similar between sedentary *db/db* mice and *db/^+^* mice (*db/db* sedentary 1.11 ± 0.38 vs. *db/^+^* sedentary 1.00 ± 0.30, *p* = 0.82). Mice of the different genotypes did, however, respond differently to HIIT, indicated by both an exercise effect (*p* = 0.02) as well as an interaction effect (*p* = 0.01): Exercise caused a 55% increase in the amount of DCX signal in the SGZ of non-diabetic *db/^+^* mice (*db/^+^* exercised 1.55 ± 0.06 vs. *db/^+^* sedentary 1.00 ± 0.30, *p* = 0.005, Figure 2a vs. 2b, 2e). Interestingly, this effect of exercise was not found in the diabetic mice; exercised *db/db* mice showed a DCX signal intensity of 1.04 ± 0.01, which was not higher than what was measured in the sedentary *db/db* mice (1.11 ± 0.38, *p* = 0.9). Additionally, we found a trend for an exercise effect between genotypes, where exercised *db/^+^* mice had 49% higher DCX signal intensity than exercised *db/db* mice (1.55 ± 0.06 vs. 1.04 ± 0.01), but this did not reach statistical significance (*p* = 0.06).

### Neurogenesis of the V-SVZ was unaffected by exercise in both diabetic and non-diabetic mice

Data from the V-SVZ did not pass the Shapiro-Wilk test of normality, hence they were log2-transformed and confirmed to be normal before the statistical analyses. Surprisingly, neither genotype nor the exercise intervention affected neurogenesis in the V-SVZ. The DCX^+^ signal in the four groups of mice were as follows: *db/^+^* sedentary 1.00 ± 0.50, *db/^+^* exercised 0.89 ± 0.22, *db/db* sedentary 0.90 ± 0.51, *db/db* exercised 0.71 ± 0.18 (Figures 2f–j), which were not statistically different from each other.

### Levels of hippocampal capillary density did not predict neurogenic levels in the SGZ

**FIGURE 3:**
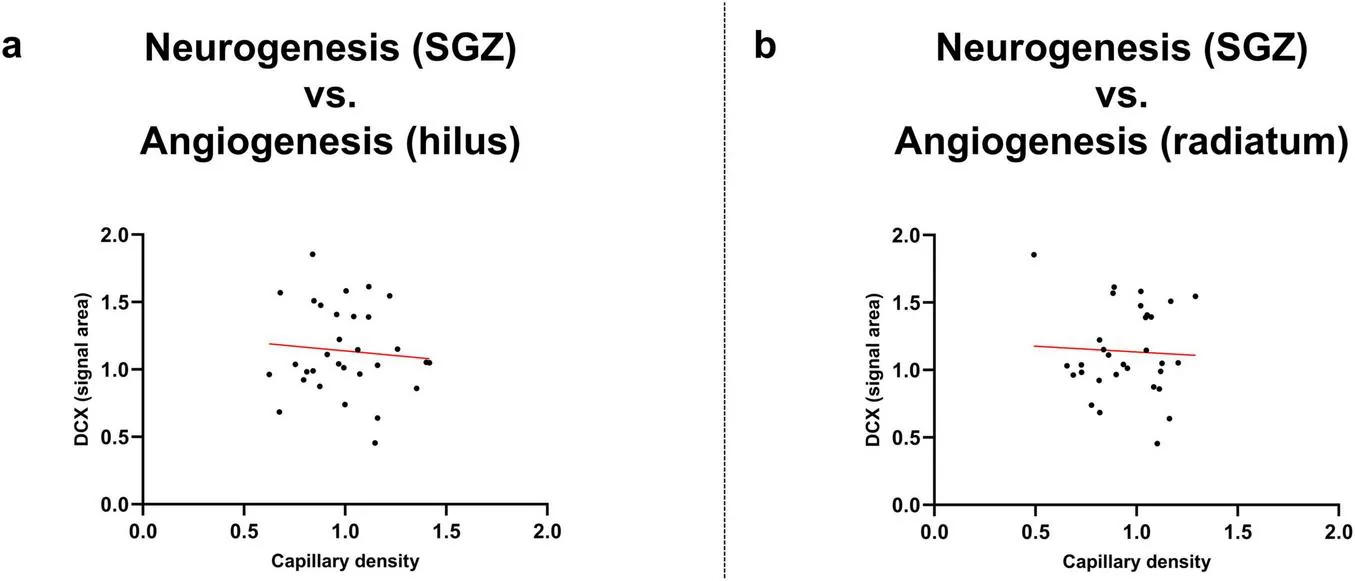
Linear regressions of hippocampal neurogenesis vs. capillary density. **(a)** Linear regression of capillary density in the hilus region (*x*-axis) vs. the doublecortin positive (DCX^+^) signal area in the subgranular zone (SGZ; *y*-axis). The dots represent the mean measure from one animal, and the solid red line represents the trendline. R^2^ = 0.008; *p* = 0.6. *n* = 31. **(b)** Linear regression of capillary density in the stratum radiatum region (*x*-axis) vs. the doublecortin positive (DCX^+^) signal area from the subgranular zone (SGZ; *y*-axis). The dots represent the mean measure from one animal, and the solid red line represents the trendline. R^2^ = 0.002; *p* = 0.8. *n* = 31.

There is a lack of consensus within the field as to whether neurogenesis is prompted by angiogenesis (Fang et al., 2023), or if these two processes occur independent of each other (Kerr et al., 2010). The fact that neurogenesis and capillary density were affected differently by exercise depending on the genotype in our mice, speaks for the latter assumption. When the DCX^+^ signal area in the SGZ was plotted against the capillary density in the hilus for each animal, we found no linear relationship (R^2^ = 0.008, *p* = 0.6; Figure 3a). Similarly, no linear relationship was found between capillary density in the stratum radiatum and the DCX^+^ signal area in the SGZ (R^2^ = 0.002, *p* = 0.8; Figure 3b).

### Open field test revealed no statistical differences in levels of anxiety between genotypes

The first 10 min of the open-field test is commonly used to assess the level of anxiety, where time spent in the center of the arena is interpreted as a sign of relaxed, explorative behavior, while time spent in corners or close to the walls of the arena reflect an instinctual fright of predators and is interpreted as a sign of stress and anxiety (Bailey, 2009; Denenberg, 1969). The non-diabetic and diabetic mice did not show a statistical difference in the time spent in the peripheral zone (median 9.0 vs. 9.6 min; *p* = 0.06, Mann-Whitney U test) (Supplementary Figure 2a). The remaining 50 min of the open-field test was used to provide a measure of the animal’s ambulatory activity. The *db/^+^* animals travelled 2.5 times longer than the *db/db* mice (44.04 ± 10.70 m vs. 16.44 ± 6.05 m; *p* < 0.0001, Supplementary Figure 2b), as expected with these genotypes.

## Discussion

In the present study we report that exercise regulates capillary density and neurogenesis differently in obese/diabetic *db/db* mice versus in non-diabetic *db/^+^* littermates. The basal levels of neurogenesis and capillary density were similar in sedentary groups of both genotypes. In response to 8 weeks of HIIT, neurogenesis in the SGZ increased in the *db/^+^* mice but not in the *db/db* mice, while capillary density in the dentate hilus increased in the *db/db* mice but not in the *db/^+^* mice. Exercise had no effect on V-SVZ neurogenesis or capillary density in stratum radiatum in either genotype. These findings challenge and nuance established concepts of cerebral angiogenesis and adult neurogenesis, and their regulation in response to T2D and physical exercise.

Given the high oxygen and glucose consumption of the brain, proper vascularization is crucial for brain health. The hippocampus has a particularly high energy expenditure which, along with a low endothelial cell-to-neuron ratio, makes it particularly sensitive to vascular dysfunction and hypoperfusion (Ventura-Antunes and Herculano-Houzel, 2022; Zhang et al., 2019). In T2D, insulin insensitivity increases the levels of circulating glucose and free fatty acids, causing lipotoxicity and inflammation (Goldin et al., 2006). Prakash et al. (2013) found higher vascularization, particularly of capillaries, in *db/db* mice compared to wild-type C57BL/6 mice in the cerebral cortex and striatum, which they interpreted as a “pathological neovascularization pattern”. This is consistent with observations in lean hyperglycemic Goto-Kakizaki T2D rats described in the same paper. In the present study the main objective was to investigate the capillary density between experimental groups, and even though the term “angiogenesis” refers purely to the formation of new blood vessels from already existing ones (Tahergorabi and Khazaei, 2012), it is important to note that the integrity of these vessels is not determined here.

Single nuclei transcriptomics of the hippocampus of male *db/db* mice (Nuthikattu et al., 2024) and male *ob/ob* mice (Milenkovic et al., 2024) also suggests that T2D negatively impacts expression of pro-angiogenic genes. In contrast to these findings, we observed no effect of the obese/diabetic phenotype *per se* on capillary density in the dentate hilus or stratum radiatum. Unlike previous studies that used C57BL/6 mice as controls, we used *db/^+^* mice to minimize genetic variation. Although *db/^+^* mice do not develop obesity/diabetes, they may not be completely phenotypically normal; for instance, female *db/^+^* mice are more prone to gestational diabetes than wildtype C57BL/6 mice (Xing et al., 2016). Hence, we cannot exclude the possibility that comparing angiogenesis in the *db/db* mice to that of wild-type (^+/+^) C57BL/6 animals would have provided different results. Additionally, Prakash et al. (2013) investigated vascularization in the cerebral cortex and striatum rather than in the hippocampus, which may also explain the discrepancy between their study and ours. Their study also did not report the sex of the animals, though a previous study from the same group used only male mice (Li et al., 2010). Similarly, the transcriptomics data mentioned above (Milenkovic et al., 2024; Nuthikattu et al., 2024) were also from male mice. The sex of the animals may be highly important for the effects of T2D on cerebral vascularization, as female mice show protection against T2D-related capillary dysfunction (Zhu et al., 2023). Our study included both male and female mice, opening for the possibility that the lack of difference between diabetic and non-diabetic mice mainly represent female resilience to T2D-induced inhibition of angiogenesis. The present study was not powered to perform analyses for each sex separately; hence we cannot rule out such sex-dependent effects.

We found that HIIT increased capillary density in the dentate hilus of *db/db* mice but not *db/^+^* mice (Figure 1). The lack of an exercise-induced effect in the dentate hilus of non-diabetic mice was surprising, as our group has previously reported exercise-induced angiogenesis in this region in wild-type C57BL/6 mice (Morland et al., 2017) with comparable exercise regimen and mouse age. Nevertheless, enhanced capillary density was observed in the *db/db* mice in response to HIIT; in fact, exercised *db/db* mice showed significantly higher capillarization than any other group. This suggests that T2D increases the brain’s angiogenic response to HIIT, possibly through shared mechanisms of T2D and HIIT, such as increased circulating Vascular endothelial growth factor A (VEGF-A) levels (Bonnefond et al., 2013; Leick et al., 2009). However, VEGF-A levels were not measured in the present study; thus, its involvement in the observed effects remains hypothetical and should be interpreted with caution. VEGF-A is a key regulator of angiogenesis and BBB function (Geiseler and Morland, 2018), and its levels have been found to be upregulated in the early stage of T2D but returning to normal at later stages (Li et al., 2021).

Manual counting of DCX-positive cells produced similar results as semi-automatic measurements of the area of the DCX signal (Supplementary Figure 1); hence, we use the latter method to conclude that the obese/diabetic phenotype *per se* did not reduce the number of neuroblasts or immature neurons. The area of DCX^+^ signal did not differ between sedentary *db/^+^* mice and sedentary *db/db* mice (Figure 2). Previous literature on whether obesity and/or T2D disrupt adult neurogenesis of the hippocampus is inconsistent. Studies on rodents have shown that neurogenesis is negatively affected by T2D (Bonds et al., 2020; Zaletel et al., 2018), and that a decline in neurogenesis is linked to a decline in cognitive flexibility. Refining the view, obesity caused by intake of a high-fat diet has been reported to inhibit neurogenesis when started at weaning but not when started in adulthood (Boitard et al., 2012). Even in the *db/db* model, results vary: Hamilton et al. (2011) found enhanced SGZ neurogenesis in response to obesity/T2D both in *db/db* and *ob/ob* mice, as well as in wild-type mice fed a high-fat diet. Nevertheless, in the same study daily subcutaneous injections of anti-diabetic GLP-1 analogues enhanced neurogenesis in all these models. On the other hand, several studies have reported reduced neurogenesis in *db/db* mice (Bracke et al., 2019; Lindqvist et al., 2006; Stranahan et al., 2008) or no difference (Rivera et al., 2011). These discrepancies may reflect the progression of diabetes/hyperglycemia. An interesting hypothesis, reviewed by Cavallucci et al. (2016), is that excessive caloric intake weakens the quiescence of neural stem cells and suppresses self-renewal. Nutrients may in themselves trigger neurogenesis, yet, as over-nutrition sustains and T2D develops, the stem cell pool may be depleted, resulting in a reduced number of immature neurons (Cavallucci et al., 2016). The lack of difference in neurogenesis between diabetic and non-diabetic mice in the present study may reflect this shift. Ramos-Rodriguez et al. (2014) observed that hippocampal neurogenesis was increased in *db/db* mice at early stages of the disease (4 weeks old), yet returned to control levels after 14 and 26 weeks, which is similar to the age of the mice used in the present study (17–19 weeks of age). Similarly, in the SVZ, neurogenesis was enhanced at 4 weeks, and then slowly decreased at 14 weeks, reaching control levels by 26 weeks of age (Ramos-Rodriguez et al., 2014).

In the present study, exercise increased the neurogenesis in the SGZ in the *db/^+^* mice (Figure 3), as expected based on previous literature (Lambertus et al., 2021, 2024; van Praag et al., 1999). The interpretation of “enhanced neurogenesis” is based on increased numbers of DCX-positive cells; as DCX does not provide information on the subsequent survival, full maturation, synaptic integration, or functional recruitment of these cells into existing circuits, our conclusions are therefore limited to early stages of neuronal development and do not establish the long-term functional impact of newly generated neurons. Other studies have, however, shown that exercise induces an increased number of newborn neurons and that these survive for several weeks, reaching a mature state (Kempermann and Gage, 1999; van Praag et al., 1999). Interestingly, we found that exercise had no neurogenic effects in the *db/db* mice. In the V-SVZ we found no effect of exercise on neurogenesis in neither genotype. This differs from our previous finding of lactate-dependent exercise-induced neurogenesis in wildtype C57BL/6 mice (Lambertus et al., 2021). Whether the lack of exercise-induced neurogenesis in the present study reflects phenotypical similarity between the *db/^+^* and *db/db* mice (discussed above), remains unresolved.

In the present study, forced exercise at high intensity was used to mimic commonly used human interval exercise regimens. Although exercise-induced neurogenesis is well-established, especially in the SGZ, the neurogenic effect of long-term HIIT is less studied. Some authors claim that forced HIIT running induces stress, which inhibits hippocampal neurogenesis (Brummelte and Galea, 2010; Lee et al., 2006), suggesting that voluntary exercise in running wheels and/or exercise at medium intensity may be more beneficial (Inoue et al., 2015). Stress is associated with elevated levels of glucocorticoids, mainly cortisol in humans and corticosterone in rodents (Svensson et al., 2016), which inhibits hippocampal neurogenesis (Numakawa et al., 2017; Planchez et al., 2020). The study by Inoue et al. (2015), often cited for this relationship, used rats rather than mice and also defined their high- and medium intensities differently. In their study, medium intensity exercise represented approximately 20 m/min and high intensity was above 20 m/min (Inoue et al., 2015). The highest speed observed in the present study was 21.7 m/min, which the *db/^+^* mice achieved only during the last intervals of the last day of exercise. Knowing that rats run slower than mice at similar VO_2max_ (Høydal et al., 2007), the “high-intensity” protocol of Inoue et al. (2015) likely brought the animals closer to exhaustion than the protocol used in the present study. Further supporting that the treadmill running in the present study was not aversively stressful, no signs of aversive behavior were observed when the mice were placed on the treadmill. Furthermore, most of the mice chose to run at low speed rather than resting on the treadmill before the exercise session. Enhanced neurogenesis in SGZ of *db^/+^* mice further indicates no stress-induced neurogenesis reduction. In the *db/db* mice, however, the situation may have been different, as these mice tended to spend more time in the periphery of the open field maze compared to the *db/^+^* mice at baseline (before the exercise intervention), indicate a tendency for increased anxiety/reduced explorative behavior (Supplementary Figure 2a). Corticosterone levels were not measured in the present study, and therefore we cannot conclude whether the *db/db* mice had a higher activation level of the hypothalamic-pituitary-adrenal axis than their *db/+* littermates in response to exercise. Nevertheless, a previous reports show that *db/db* mice have elevated plasma levels of corticosterone, the predominant glucocorticoid in mice, (Erickson et al., 2017, Physiol. Behav) and that this contributes to the impairment of synaptic plasticity and neurogenesis, and associated learning and memory deficits (Stranahan et al., 2008), and higher anxiety than *db/^+^* mice (Dinel et al., 2011). In line with this, human patients with T2D show higher anxiety and higher levels of the stress hormone cortisol (Champaneri et al., 2010; Chiodini et al., 2005) compared to non-diabetic people. Studies also suggest that the cortisol levels and hyperglycemia mutually enhance each other, forming a vicious cycle (Joseph and Golden, 2017). Hence, it is not unlikely that the *db/db* mice, despite being exercised at a speed adjusted to their endurance capacity, experienced greater stress than their *db/^+^* littermates and that this may explain their lack of HIIT-induced neurogenesis.

In conclusion, the present study shows that 8 weeks of HIIT enhanced capillary density in the dentate hilus of diabetic *db/db* mice, while the hilar capillary density of non-diabetic mice was unaffected by HIIT. On the other hand, neurogenesis in the SGZ was increased by exercise in non-diabetic mice but remained unaffected by exercise in their diabetic littermates. No effect of exercise or genotype were observed on capillary density in the stratum radiatum or neurogenesis in the V-SVZ. These findings suggest that T2D alters the effects of exercise on the adult brain. Understanding these differential responses provide insights into the cerebral effects of diabetes and could pave the way for customized interventions to improve brain health in individuals with metabolic disorders.

## Data Availability

The raw data supporting the conclusions of this article will be made available by the authors, without undue reservation.

## Footnotes

1
http://www.med.uio.no/imb/english/research/about/infrastructure/norbrain/slidescanning/

## Supplementary material

Supplementary Figure 1 Linear regression model comparing manual counting of doublecortin positive (DCX^+^) cells and the DCX^+^ signal area (signal area/μm). The dots represent the mean measure from one animal, and the solid red line represents the trendline. R^2^ = 0.68; *p* < 0.0001. Number of animals (n): 31 mice.

Supplementary Figure 2 Open field test (OFT) of mice prior to the exercise regime. **(A)** Bar plot showing the mean time in minutes spent in the peripheral zone by *db/^+^* mice (light gray bars) and *db/db* mice (dark grey bars). Dots represent observations from individual animals. Error bars indicate standard deviation (SD). Number of animals (n): 21 *db/^+^*, 16 *db/db*. **(B)** Bar plot showing the mean distance travelled in meters (m) by *db/^+^* mice (light gray bars) and *db/db* mice (dark grey bars). Dots represent observations from individual animals. Error bars indicate SD. n: 19 *db/^+^*, 16 *db/db*. *****p* < 0.0001.
